# Supplementary material for: PAM-repeat associations and spacer selection preferences in single and co-occurring CRISPR-Cas systems
Source: Genome Biol. 2021 Sep 30;22:281. doi: 10.1186/s13059-021-02495-9 (PMC8482600; doi:10.1186/s13059-021-02495-9)
Supplement: Supplementary file 1 — Additional file 1. Supplementary figures. [file 13059_2021_2495_MOESM1_ESM.docx]

# Supplementary figures


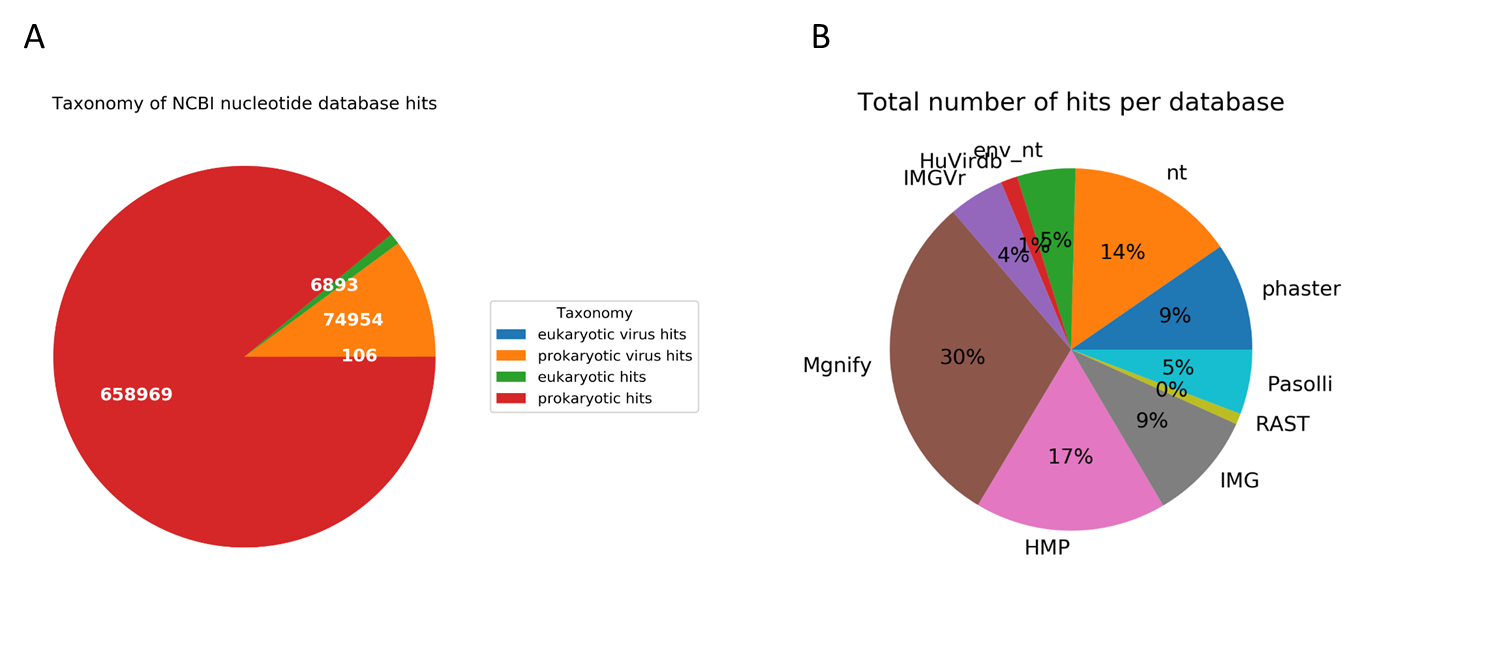


**Supplementary figure 1. Taxonomy of spacer targets and number of found targets per database.** (A) The taxonomy of targeted sequences of the NCBI nucleotide database was obtained from the NCBI taxonomy database. For hits in viral sequences, the taxonomy of known hosts was used to label the virus as a eukaryotic or prokaryotic virus. (B) The contribution of each database to the total number of hits after filtering. All databases were accessed in February 2020.


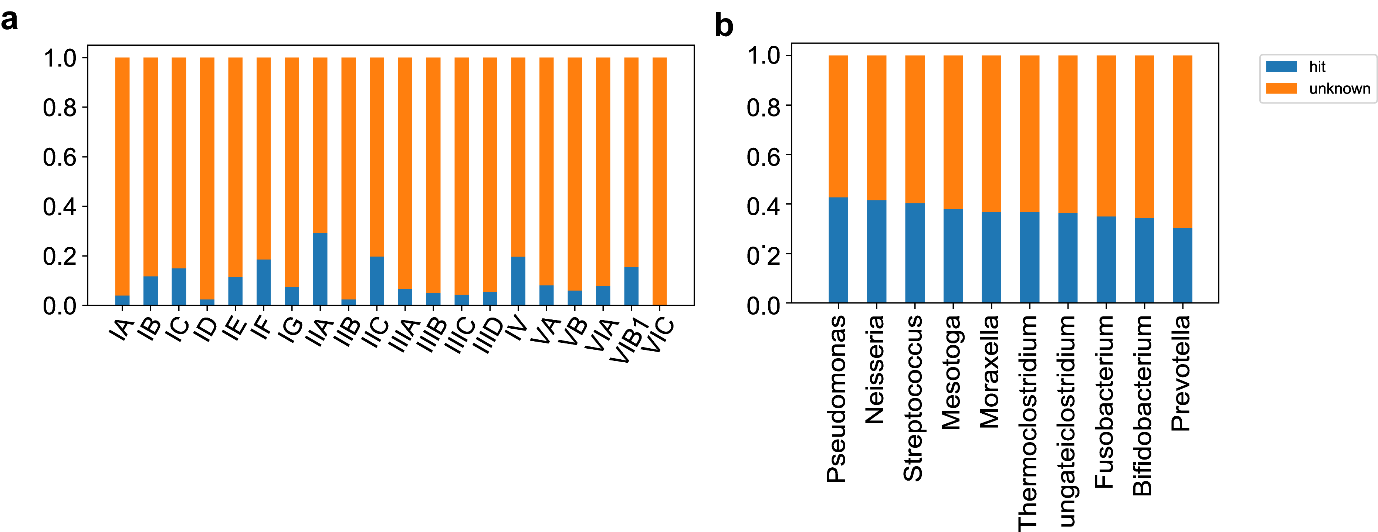


**Supplementary figure 2. Perfect match statistics.** (A) Distribution of fraction of hits per subtype as in Figure 1G but only for perfect matching (100% identity) spacers. (B) Distribution of fraction of hits for the ten highest scoring genera as in Figure 1E (with at least 500 spacers) but only for perfect matching spacers.

­­
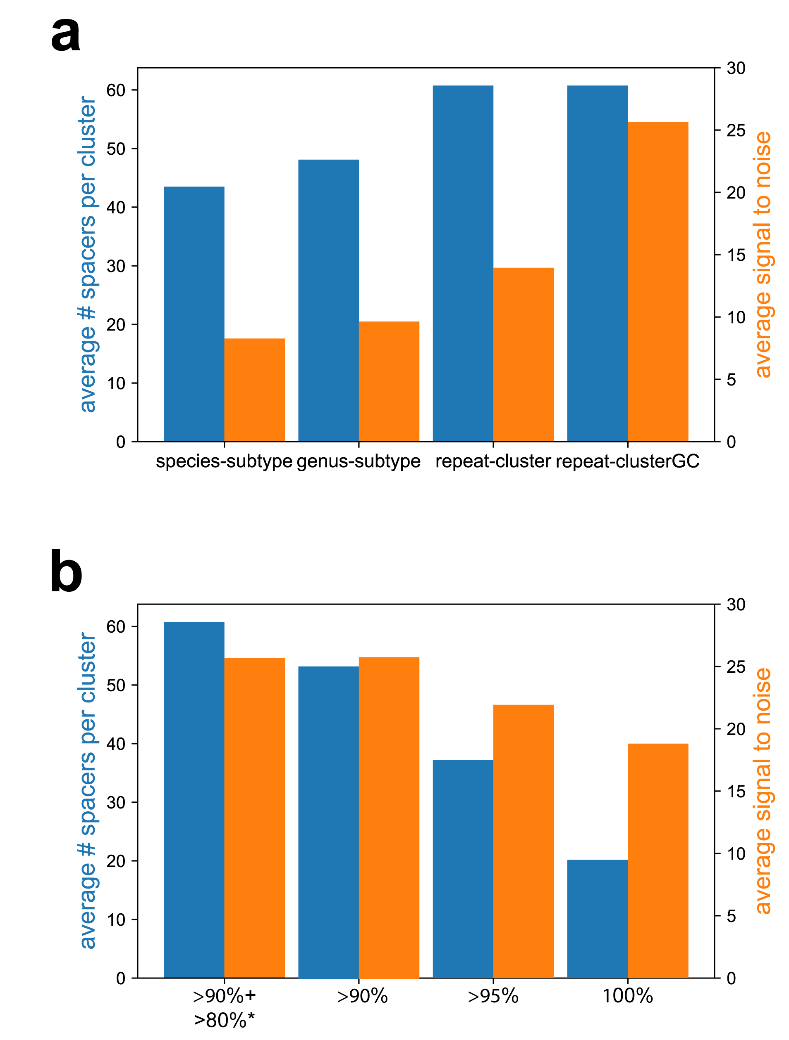


**Supplementary figure 3. Average number and signal-to-noise ratio of clustered hits.** Different clustering methods were compared for their average number of unique hits (blue) and average signal-to-noise ratio (orange). The signal-to-noise ratio was calculated by dividing the average information content of the two top positions in the flank (potential PAM nucleotides) by the median information content in sequence logos generated from flanks of hits. (B) The clustering categories are based on whether spacers come from same species and subtype (species-subtype), from same genus and subtype (genus-subtype), from clusters of repeat sequences with 90% identity (repeat-cluster) or clusters of repeat sequences with 90% identity and additionally compensation for GC-content of spacers within the cluster (see Materials and Methods, repeat-clusterGC). (B) The average number of hits and signal-to-noise ratio in case of using increasing levels of nucleotide identity to filter hits. The >80%* indicates hits that have >80% identity but they are only accepted in case a different spacer within the same genus targets the same sequence.


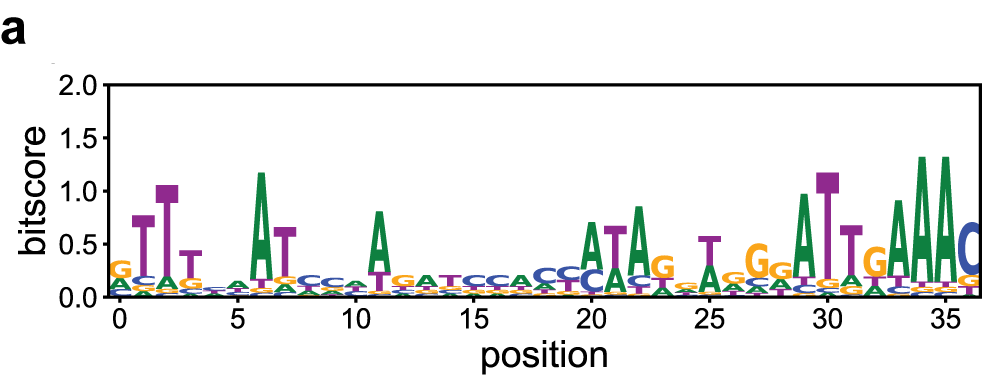


**Supplementary figure 4. Sequence logo Type III repeats**. ClustalW alignment of Type III repeats for which orientation was determined based on presence of PAM (n = 21 unique repeats). The 3'end of the repeat, which is the 5' handle of the transcribed crRNA, has a conserved motif (ATTGAAAC).


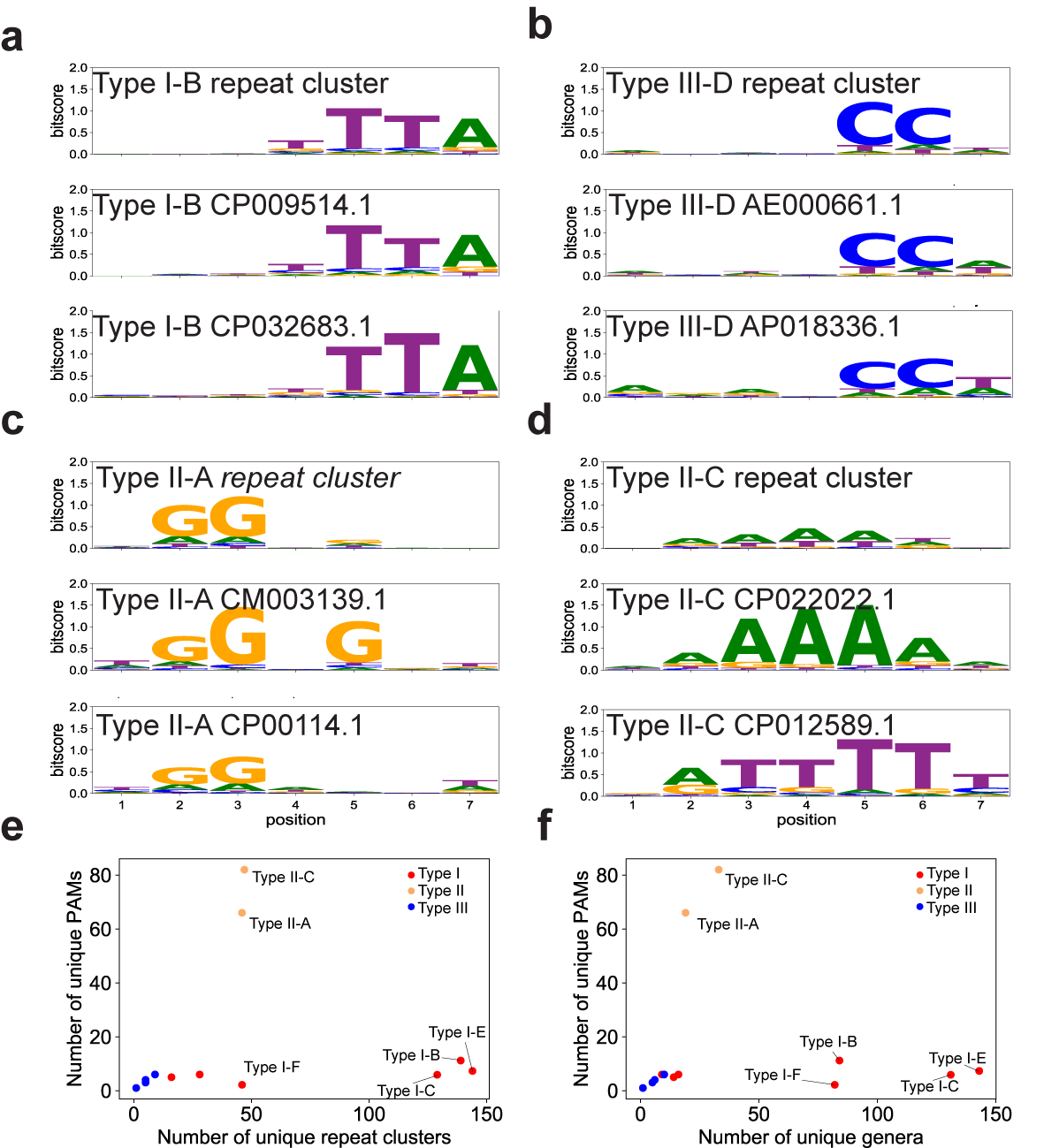


**Supplementary figure 5. Sequence logo of protospacer flanking regions of Type I and Type II systems based on clustering approach and PAM diversity plots for each subtype**. For each subtype ((A) Type I-B; (B) Type III-D; (C) Type II-A and (D) Type II-C) a representative cluster was chosen and a sequence logo was made as in Figure 2 for either all spacers within that cluster (top), or two representative genomes within that cluster (middle, bottom). For Type I and Type III system, the PAM is conserved within a repeat cluster (A) and (B) whereas for Type II systems, the PAM can differ within a cluster (C,D). (E-F) The number of unique PAMs found for each subtype depending on (E) the number of unique repeat clusters for each subtype and (F) the number of unique genera within each subtype for Type I (red), Type II (orange) and Type III (blue) systems.


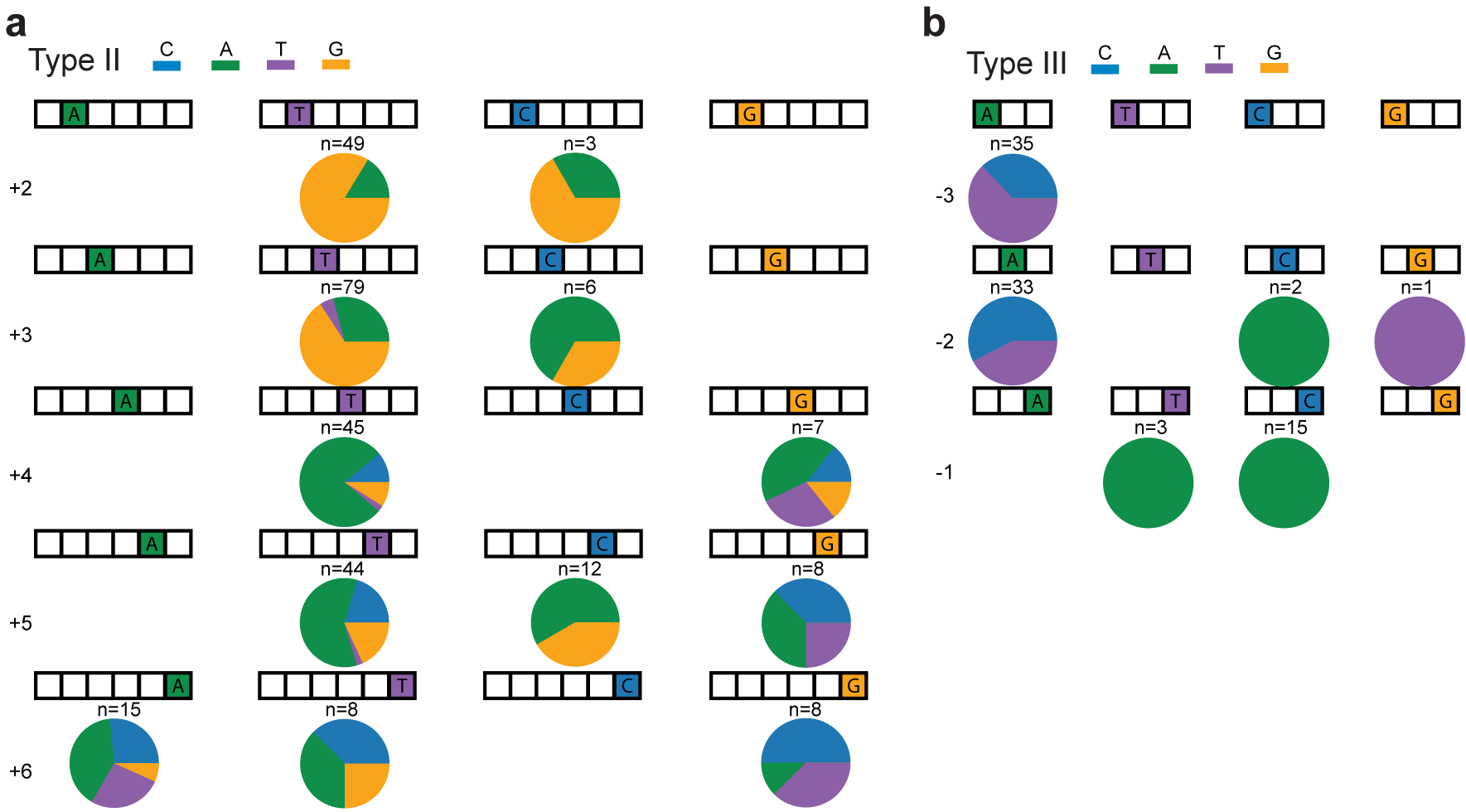
 **Supplementary Figure 6. Relationship between repeat and PAM sequence of Type II and Type III systems.** Same as Figure 3B except for Type II (A) and Type III (B) systems
